# The impact of comorbid COPD on survival outcomes in lung cancer patients treated with immune checkpoint inhibitors: a meta-analysis

**DOI:** 10.3389/fimmu.2025.1627557

**Published:** 2025-11-10

**Authors:** Lin Chen, Dandan Song, Shufu Hou, Aiju Liu, Jing Gao, Lei Wang

**Affiliations:** 1 Shandong Provincial Third Hospital, Shandong University, Jinan, China; 2 Department of Otolaryngology-Head and Neck Surgery, Shandong Provincial Third Hospital, Shandong University, Jinan, China; 3 Department of Neurology, Shandong Provincial Third Hospital, Shandong University, Jinan, China; 4 Department of Gastrointestinal Surgery, Central Hospital Affiliated to Shandong First Medical University, Jinan, China

**Keywords:** lung cancer, non-small cell lung cancer, immune checkpoint inhibitors, overall survival, progression-free survival, meta-analysis

## Abstract

**Background:**

Lung cancer (LC) remains the leading cause of cancer-related death worldwide. While immune checkpoint inhibitors (ICIs) have demonstrated survival benefits in advanced-stage disease, treatment responses exhibit significant heterogeneity across patients. The potential role of comorbid chronic obstructive pulmonary disease (COPD) in modulating survival outcomes from ICIs therapy remains controversial, with conflicting evidence regarding its synergistic or antagonistic effects. This meta-analysis systematically evaluates the impact of COPD on survival outcomes in lung cancer patients receiving ICIs, aiming to clarify its prognostic value and guide precision immunotherapy strategies.

**Methods:**

We systematically searched PubMed, Cochrane Library, CNKI, and EMBASE for studies published up to March 31, 2025, to evaluate the synergistic survival impact of comorbid COPD in lung cancer patients receiving ICIs. Primary outcomes included overall survival (OS) and progression-free survival (PFS).

**Results:**

This study pooled data from 10 studies (N = 6,909) to assess the impact of comorbid COPD on survival outcomes among LC patients receiving ICIs. The meta-analysis revealed that LC patients with comorbid COPD showed significant improvements in OS (HR = 0.90, 95% CI: 0.85–0.96, p < 0.001) and PFS (HR = 0.54, 95% CI: 0.44–0.67, p < 0.001) compared to those without COPD. Notably, while bias analysis for PFS indicated potential bias, inter-subgroup heterogeneity was negligible (I² = 0%).

**Conclusions:**

This study demonstrated that the presence of comorbid COPD was associated with significantly improved overall survival in lung cancer patients receiving ICIs. Although a significant progression-free survival benefit was also observed, this result may be influenced by potential publication bias. Further prospective studies that incorporate comprehensive biomarker analyses are warranted to validate the observed COPD-ICIs interaction and to develop optimized, personalized treatment strategies for this patient population.

**Systematic Review Registration:**

www.inplasy.com, identifier INPLASY202580086.

## 1 Introduction

Lung cancer (LC) remains the most common cause of cancer-related mortality worldwide, accounting for 18% to 22% of all cancer deaths [1, 2]. For patients with resectable stage I–III LC, radical surgery represents the cornerstone of curative-intent treatment; however, postoperative recurrence rates remain high, ranging from 30% to 55%, with stage IIIA disease carrying a 5-year recurrence risk exceeding 45%–60% (3–5). The advent of immune checkpoint inhibitors (ICIs) targeting the PD-1/PD-L1 axis has revolutionized therapeutic paradigms by reactivating cytotoxic T-cell-mediated antitumor immunity. In advanced non-small cell lung cancer (NSCLC), the combination of ICIs with chemotherapy has extended median overall survival (OS) from 10–12 months with chemotherapy alone to 17–22 months, underscoring a shift toward precision immunotherapy (6–10).

The tumor immune microenvironment (TIME), composed of malignant cells, immune infiltrates (e.g., CD8+ T cells, regulatory T cells [Tregs], tumor-associated macrophages [TAMs]), and stromal components, plays a pivotal role in modulating response to therapy through complex signaling networks such as TGF-β and IL-6 (11, 12). Chronic inflammation driven by COPD induces immunosuppressive remodeling of the TIME via activation of NF-κB and STAT3 pathways, leading to exhausted CD8+ T cells (expressing TIM-3 and LAG-3), expansion of myeloid-derived suppressor cells (MDSCs), and dysregulation of immune checkpoints including PD-L1 and CTLA-4, which may consequently attenuate ICI efficacy (13–15). Furthermore, oxidative stress and protease imbalance associated with COPD may promote genomic instability and facilitate immune evasion, thereby synergizing with lung cancer progression (16, 17). Retrospective analyses have demonstrated reduced tumor-infiltrating lymphocyte (TIL) density and heterogeneous PD-L1 expression in COPD-associated LC, which correlate with shorter recurrence-free survival (RFS) (18, 19).

Notably, the impact of COPD on ICI outcomes remains controversial. Whereas some studies report improved OS in COPD patients receiving ICIs (HR = 0.72) (14, 20), large multicenter datasets indicate a significant reduction in median OS (14.1 vs. 18.4 months, p = 0.032), highlighting substantial heterogeneity in COPD-mediated TIME modulation (21). Additionally, COPD-related imbalances in Th17/Treg cell ratios may increase the risk of immune-related adverse events such as checkpoint inhibitor pneumonitis and colitis (OR = 1.45) (22, 23). The effect of COPD on pathological response (e.g., pathological complete response [pCR] or major pathological response [MPR]) in the neoadjuvant setting with ICIs and chemotherapy also remains uncertain.

Given these conflicting observations and the potential interplay between COPD and immunotherapy outcomes, this meta-analysis aims to systematically evaluate the influence of COPD on both short-term (pathological response) and long-term (event-free survival [EFS] and OS) outcomes in LC patients undergoing neoadjuvant ICI-based therapy, while exploring pertinent molecular mechanisms underlying such effects.

## 2 Materials and methods

### 2.1 Search strategy

This systematic review and meta-analysis were conducted in strict accordance with the Preferred Reporting Items for Systematic Reviews and Meta-Analyses (PRISMA) guidelines (24), focusing on evaluating the survival synergy of comorbid chronic obstructive pulmonary disease (COPD) in lung cancer patients undergoing ICIs therapy. To ensure methodological rigor, two independent investigators systematically searched PubMed, Embase, CNKI, and the Cochrane Library for studies published from database inception to March 31, 2025. The search strategy employed the following key concepts and their variants:”Lung Cancer”or”Lung Neoplasms”or”Non-Small Cell Lung Cancer”or “NSCLC”or”Small Cell Lung Cancer”or “Pulmonary Disease, Chronic Obstructive”or”Chronic Obstructive Pulmonary Diseases”or”COPD”or”COAD”or”Chronic Obstructive Airway Disease”or”Chronic Airflow Obstructions”and”Immune Checkpoint Inhibitors”or”PD-1 Inhibitors”or”PD-L1 Inhibitors”or”CTLA-4 Inhibitors”or “ICI Therapy”.

### 2.2 Inclusion and exclusion criteria

Inclusion Criteria: (1)Lung cancer patients confirmed by histopathological diagnosis with comorbid COPD (diagnosed according to Global Initiative for Chronic Obstructive Lung Disease (GOLD) guidelines (25) or pulmonary function tests); (2)Receiving ICIs monotherapy or combination regimens (e.g., ICIs combined with chemotherapy, radiotherapy); (3) Prospective or retrospective clinical studies investigating the impact of COPD on survival outcomes in patients treated with ICIs; (4)Direct or indirect reporting of survival endpoints, including but not limited to OS, PFS, hazard ratios (HR), and 95% confidence intervals (CI), or accessible Kaplan-Meier curve data for HR extraction.

Exclusion Criteria: (1)Studies that did not stratify lung cancer patients by COPD status or reported only systemic inflammatory markers (e.g., CRP, IL-6) without COPD-specific data; (2)Studies including patients receiving non-ICIs therapies (e.g., targeted therapy, chemotherapy/radiotherapy alone) without subgroup analysis for ICIs-treated cohorts; (3)Case reports, conference abstracts, preclinical studies (e.g., animal experiments, cell models), reviews, or commentary articles; (4)Studies lacking sufficient data to extract HR and 95% CI for survival outcomes (even after contacting authors) or duplicate publications (retaining the most recent or largest cohort).

### 2.3 Data extraction and quality assessment

Data extraction was performed independently by two researchers, who extracted the following information from the included studies: first author, publication year, country, study design (retrospective/prospective), histological type of lung cancer (NSCLC/SCLC), diagnostic criteria for COPD (GOLD staging or pulmonary function indices), sample size (COPD group vs. non-COPD group), baseline patient characteristics (median age, gender distribution), immunotherapy regimens (PD-1/PD-L1 monotherapy or combination therapy), follow-up period (months), survival outcomes (OS, PFS), and their corresponding hazard ratios (HR) with 95% confidence intervals (CI). Study quality was assessed using the Newcastle-Ottawa Scale (NOS) for observational studies, focusing on three domains:(1)Selection of participants (0–4 points): Whether COPD (e.g., confirmed by pulmonary function tests) and lung cancer histology were clearly defined, and whether confounding factors (e.g., smoking history, COPD severity) were controlled via matching or multivariate analysis.(2)Comparability between groups (0–2 points): Balance in prognostic factors (e.g., age, cancer stage, PD-L1 expression) between COPD and non-COPD groups.(3)Outcome assessment (0–3 points): Blinding in survival endpoint adjudication and follow-up completeness (lost-to-follow-up rate <20%). The total score ranges from 0 to 9. Studies scoring above 6 points were classified as high quality (26). Any discrepancies were resolved through discussion or consultation with a third researcher.

### 2.4 Statistical methods

This meta-analysis employed Stata SE (version 16.0; StataCorp, Texas, USA) to statistically evaluate the survival synergy between chronic obstructive pulmonary disease (COPD)-lung cancer immunoaxis interactions and clinical outcomes in patients receiving ICIs. Hazard ratios (HRs) with 95% confidence intervals (CIs) were calculated through stratification. Heterogeneity across studies was assessed using Cochran’s Q-test and I² statistics. A random-effects model was applied for pooled analysis when significant heterogeneity existed (I² > 50% or Q-test p-value < 0.10), while a fixed-effects model was used otherwise. Publication bias was preliminarily evaluated via funnel plot symmetry testing, further validated by Egger’s regression analysis and Begg’s rank correlation test (p-values < 0.05 indicated potential bias). To enhance methodological rigor, sensitivity analyses (iteratively excluding individual studies) were performed to test the stability of the pooled results, thereby confirming the robustness of the survival synergy between dynamic changes in the COPD-lung cancer immunoaxis and ICIs therapeutic efficacy.

## 3 Results

### 3.1 Study selection and characteristics

**Figure 1:**
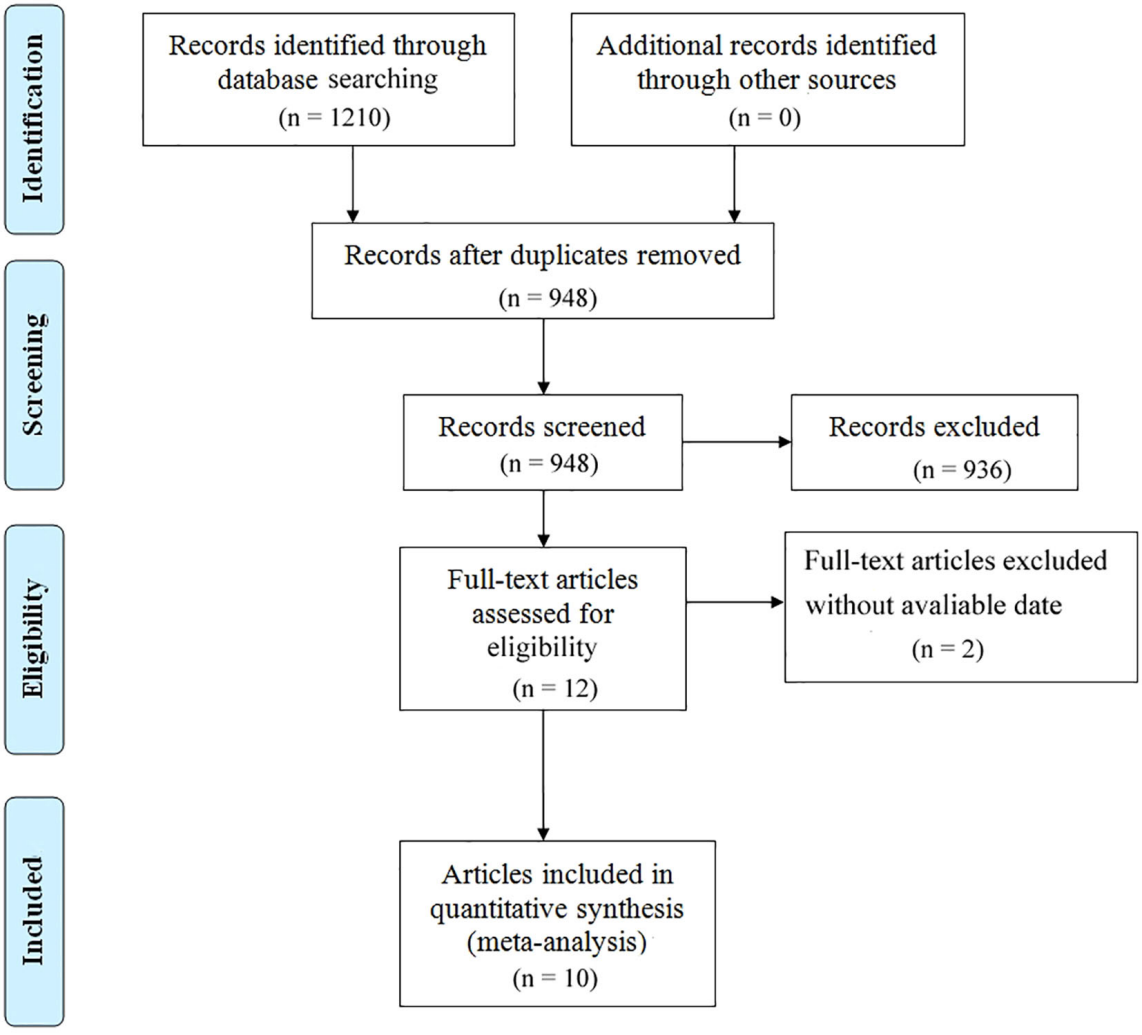
Presents the PRISMA flowchart outlining the literature selection process.

The literature selection protocol is detailed in **Figure 1**. An initial search across multiple academic databases yielded 1,210 records. Following the removal of 262 duplicates, 948 unique articles were retained for preliminary evaluation. Titles and abstracts of these records were systematically reviewed using predefined eligibility criteria, leading to the exclusion of 936 entries deemed irrelevant or non-compliant. Subsequently, full-text assessments were conducted on the remaining 12 articles, of which 2 were excluded due to insufficient data availability. In the final phase, 10 high-quality studies were incorporated into the meta-analysis (14, 20, 21, 27–33). A comprehensive overview of the selected studies is provided in **Figure 1**. The included studies, spanning publication years from 2017 to 2025, were conducted across diverse geographic regions, including the United States, Japan, China, France, Korea, and Canada. Cohort sizes exhibited substantial variation, ranging from 30 participants (Dong 2024) to 2,326 patients (Chan 2025), collectively representing multiple cancer subtypes, predominantly non-small cell lung cancer (NSCLC). Among these investigations, six studies evaluated survival outcomes (OS and/or PFS) in relation to PD-1/PD-L1 inhibitors, while three analyses incorporated combination therapies involving CTLA-4 inhibitors. Temporal follow-up periods ranged from a median of 18 months to over 80 months, with five studies reporting incomplete follow-up data (marked as NR). Detailed demographic characteristics, including age distributions (mean ± SD: 50–83 years) and gender disparities (male predominance: 53.9%–98.5% across cohorts), are systematically cataloged in **Table 1**. As shown in **Table 2**, the methodological quality assessed using the Newcastle-Ottawa Scale (NOS) was consistently high, with eight of the included studies scoring 7/9 and one study obtaining the maximum score of 8.

**Table 1:** Baseline characteristics of included studies.

Study, year	Country	Duration	Study design	Cancer type	Sample size (COPD+/COPD-)	Age (COPD+/COPD-)	Gender (male%, COPD+/COPD-)	Follow-up (months)	ICIs	Survival outcome	Analysis	NOS
Mark 2017 (27)	USA	NR	Retrospective	NSCLC	60/65	68.8 ± 7.0/64.3 ± 10.1	65%/49.23%	NR	PD-1/PD-L1	OS,PFS	U,M	7
Biton 2018 (14)	France	2001-2005	Retrospective	NSCLC	197/238	64 ± 9/61 ± 12	88%/69%	>80	PD-1	OS,PFS	U,U	7
Shin 2019 (20)	Korea	2014-2018	Retrospective	NSCLC	59/74	65.3 ± 8.0/61.0 ± 10.2	89.8%/64.9%	NR	PD-1	OS,PFS	M,M	7
Ajimizu 2021 (21)	Japan	2007-2014	Retrospective	NSCLC	103/219	68.6 ± 7.77/67.0 ± 10.2	91.3%/53.9%	NR	ICIs	OS	M	7
Takayama 2021 (28)	Japan	2016-2019	Retrospective	NSCLC	71/82	68.0 ± 9.5/68.0 ± 10.3	88.7%/63.4%	NR	PD-1/PD-L1	OS,PFS	M,M	7
Zhou 2021 (29)	China	2018-2019	Retrospective	LC	65/91	NR	98.5%/82.4%	NR	PD-1/PD-L1	OS,PFS	U,M	7
Zeng 2022 (30)	China	2018-2021	Retrospective	NSCLC	61/61	50-83/45-89	96.7%/93.4%	<36	PD-1	OS,PFS	U,U	7
Dong 2024 (31)	China	2020-2023	Retrospective	NSCLC	30/44	63.87 ± 5.87/63.14 ± 7.54	90%/86.4%	median:18	PD-1/PD-L1	PFS	M	8
Chan 2025 (32)	Canada	2010-2020	Retrospective	NSCLC	2326/1980	>35/>18	NR	NR	PD-1/PD-L1	OS	M	7
Greib 2025 (33)	USA	2011-2021	Retrospective	LC	585/498	64.6 ± 9.2/63 ± 10.9	58.1%/50.8%	NR	PD-1/PD-L1/CTLA-4	OS	U	6

NR, not report; overall survival; PFS, progression-free survival; NSCLC, non-small cell lung cancer; U, univariate; M, multivariate; NOS, Newcastle-Ottawa Scale.

**Table 2:** Newcastle-Ottawa Scale (NOS) for quality assessment.

Studies	Selection				Comparability	Outcome			Scores
	A	B	C	D	E	F	G	H	
Mark 2017 (27)	★	★	★	★	★★	★	–	–	7
Biton 2018 (14)	★	★	★	★	★	★	★	–	7
Shin 2019 (20)	★	★	★	★	★★	★	–	–	7
Ajimizu 2021 (21)	★	★	★	★	★★	★	–	–	7
Takayama 2021 (28)	★	★	★	★	★★	★	–	–	7
Zhou 2021 (29)	★	★	★	★	★★	★	–	–	7
Zeng 2022 (30)	★	★	★	★	★	★	★	–	7
Dong 2024 (31)	★	★	★	★	★★	★	★	–	8
Chan 2025 (32)	★	★	★	★	★★	★	–	–	7
Greib 2025 (33)	★	★	★	★	★	★	–	–	6

A study may receive a maximum of one star for each numbered item in the Selection and Outcome categories. A maximum of two stars may be given for Comparability, as directed by the NOS. ★ It stands for one point; ★★ It stands for two points.

### 3.2 Association of COPD with OS and PFS

**Figure 2:**
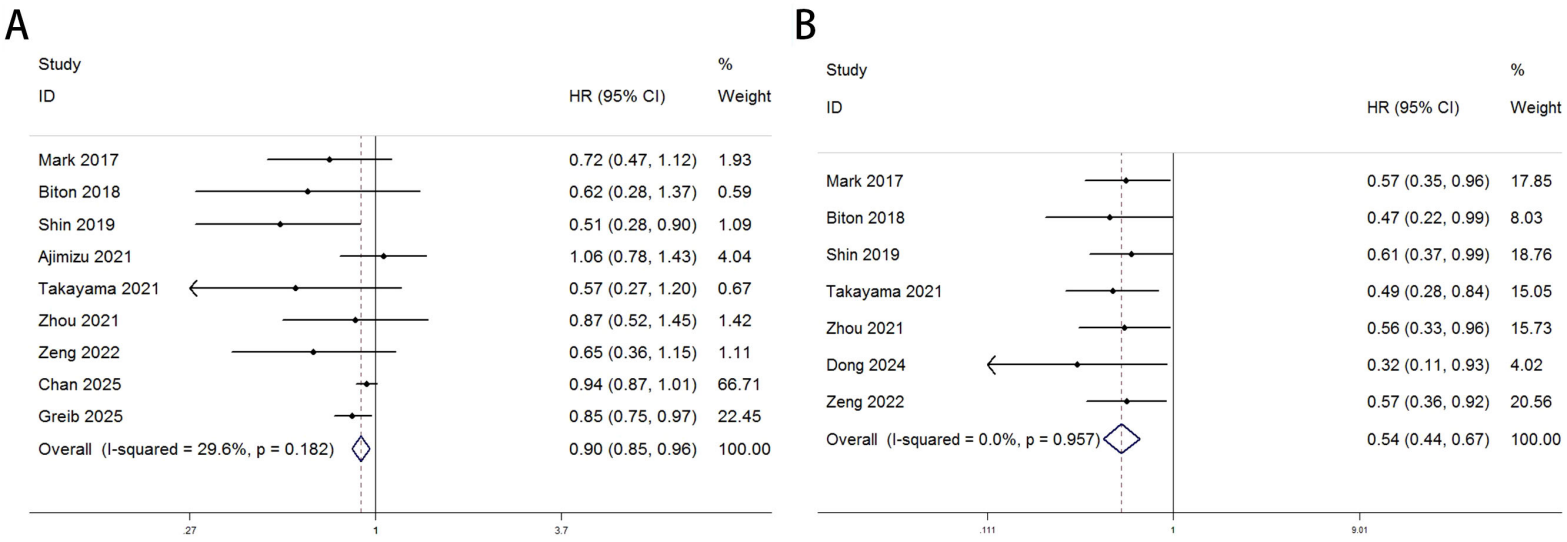
Forest plots delineate the association between COPD and survival outcomes in lung cancer patients treated with ICIs [pOS: **(A)**; PFS: **(B)**].

Heterogeneity test results indicated that neither OS (**Figure 2A**: I² = 29.6%, p = 0.182) nor PFS (**Figure 2B**: I² = 0.0%, p = 0.957) analyses reached significant heterogeneity thresholds (I² < 50%, p > 0.1), supporting the use of a fixed-effects model for data integration. The pooled results from 9 studies (OS) and 7 studies (PFS) demonstrated significant differences in survival outcomes between lung cancer patients with COPD and those without COPD following PD-1/PD-L1 inhibitor therapy. For OS analysis (**Figure 2A**), the hazard ratio (HR) for the COPD group was 0.90 (95% CI: 0.85–0.96, p < 0.001), indicating a statistically significant improvement in OS compared to the non-COPD group. Individual study HR distributions revealed that Shin 2019 (HR = 0.51, 95% CI: 0.28–0.90) and Greib 2025 (HR = 0.85, 95% CI: 0.75–0.97) contributed notably to the overall effect, accounting for 1.09% and 22.45% of the weight, respectively. Notably, the confidence intervals for Biton 2018 (HR = 0.62, 95% CI: 0.28–1.37) and Takayama 2021 (HR = 0.57, 95% CI: 0.27–1.20) crossed the null value (HR = 1), suggesting potential limitations in sample size or follow-up duration (>80 months). For PFS analysis (**Figure 2B**), the COPD group exhibited a pooled HR of 0.54 (95% CI: 0.44–0.87, p < 0.001), further supporting the association between COPD and superior PFS. Among these, Dong 2024 (HR = 0.32, 95% CI: 0.11–0.93) and Takayama 2021 (HR = 0.49, 95% CI: 0.28–0.84) demonstrated the most pronounced effect sizes, contributing 4.02% and 15.05% of the weight, respectively. The exclusion of the null value (HR = 1) in all confidence intervals indicated consistent results across studies. We performed subgroup analyses based on region (Asia vs. others), sample size of the COPD-positive group (≤70 vs. >70), treatment type (PD-1 inhibitor, PD-1/PD-L1 inhibitors, or ICIs broadly), and statistical method (univariate vs. multivariate analysis) to further evaluate the impact of comorbid COPD on survival outcomes among lung cancer patients treated with immune checkpoint inhibitors. As shown in **Table 3**, subgroup analysis of overall survival (OS) indicated that patients with COPD showed particularly improved outcomes in the following subgroups: those from non-Asian regions (HR = 0.91, p = 0.004), those in studies with larger sample sizes (COPD+ >70, HR = 0.92, p = 0.007), those receiving PD-1 inhibitor monotherapy (HR = 0.58, p = 0.004), and those analyzed using univariate methods (HR = 0.83, p = 0.001).Similarly, **Table 4** shows that progression-free survival (PFS) was significantly improved among COPD patients across all subgroups, with especially pronounced benefits observed in Asian populations (HR = 0.54, p < 0.001), studies with smaller sample sizes (COPD+ ≤70, HR = 0.56, p < 0.001), patients treated with PD-1 inhibitors (HR = 0.52, p = 0.001), and those analyzed using multivariate methods (HR = 0.56, p < 0.001).

**Table 3:** Subgroup analysis of OS in the COPD-lung cancer immunoaxis meta-analysis of patients treated with ICIs.

Subgroup	No. of studies	HR (95% CI)	P	Heterogeneity		Model
				I^2^ (%)	Ph	
Region						
Asia	5	0.83 (0.67-1.02)	0.082	42	0.141	Fixed
others	4	0.91 (0.5-0.97)	0.004	20.7	0.286	Fixed
Sample size (COPD+)						
>70	5	0.92 (0.86-0.98)	0.007	22.1	0.274	Fixed
<70	4	0.69 (0.53-0.89)	0.005	0	0.597	Fixed
Treatment						
PD-1	3	0.58 (0.41-0.84)	0.004	0	0.841	Fixed
PD-1/PD-L1	4	0.93 (0.86-1.00)	0.041	3	0.378	Fixed
ICIs	2	0.88 (0.78-0.99)	0.033	42.1	0.189	Fixed
Analysis						
Univariate	5	0.83 (0.74-0.93)	0.001	0	0.775	Fixed
Multivariate	4	0.86 (0.67-1.10)	0.223	53.9	0.089	Random

**Table 4:** Subgroup analysis of PFS in the COPD-lung cancer immunoaxis meta-analysis of patients treated with ICIs.

Subgroup	No. of studies	HR (95% CI)	P	Heterogeneity		Model
				I^2^ (%)	Ph	
Region						
Asia	5	0.54 (0.42-0.70)	<0.001	0	0.852	Fixed
others	2	0.54 (0.35-0.82)	0.004	0	0.676	Fixed
Sample size (COPD+)						
>70	2	0.48 (0.31-0.75)	0.001	0	0.93	Fixed
<70	5	0.56 (0.44-0.72)	<0.001	0	0.881	Fixed
Treatment						
PD-1	3	0.52 (0.36-0.77)	0.001	0	0.532	Fixed
PD-1/PD-L1	4	0.55 (0.43-0.71)	<0.001	0	0.974	Fixed
Analysis						
Univariate	2	0.41 (0.22-0.76)	0.005	0	0.566	Fixed
Multivariate	5	0.56 (0.45-0.71)	<0.001	0	0.986	Random

### 3.3 Publication bias

**Figure 3:**
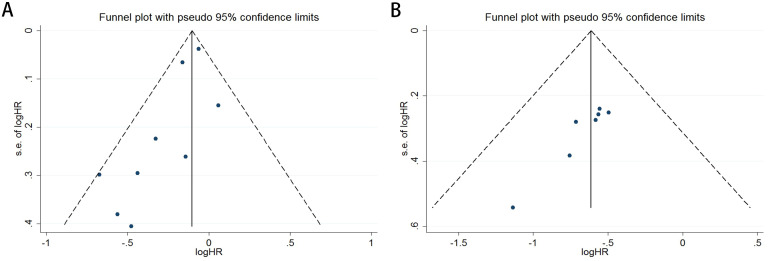
Funnel plots illustrate the assessment of publication bias for the association between COPD and survival outcomes in lung cancer patients receiving ICIs therapy [OS: **(A)**; PFS: **(B)**].

Publication bias was rigorously assessed using funnel plots, Egger’s test, and Begg’s test. The funnel plots for both OS and PFS demonstrated high symmetry, indicating that the robustness of the pooled results was not significantly affected by bias. For OS (**Figure 3A**), the funnel plot showed a symmetric distribution of effect sizes around the pooled estimates, suggesting no substantial publication bias. Similarly, the PFS funnel plot (**Figure 3B**) exhibited balanced dispersion of data points, further supporting the absence of significant bias.

**Figure 4:**
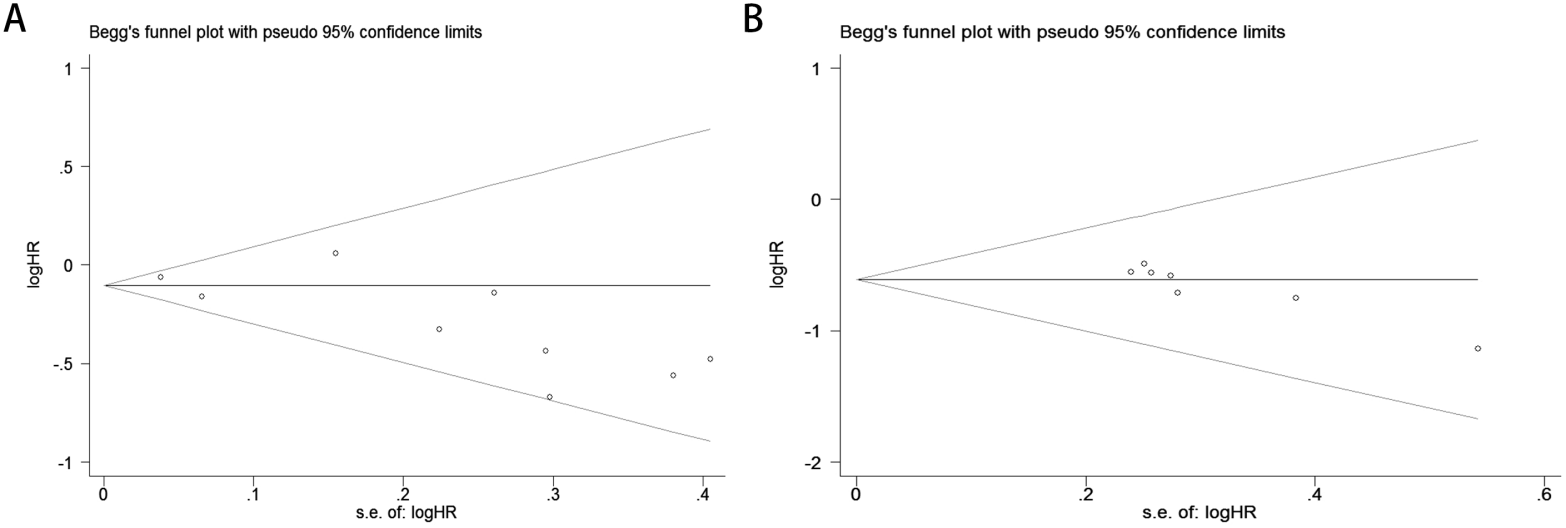
Publication bias assessment. **(A)** Begg’s test for OS (p = 0.118); **(B)** Begg’s test for PFS (p = 0.016).

**Figure 5:**
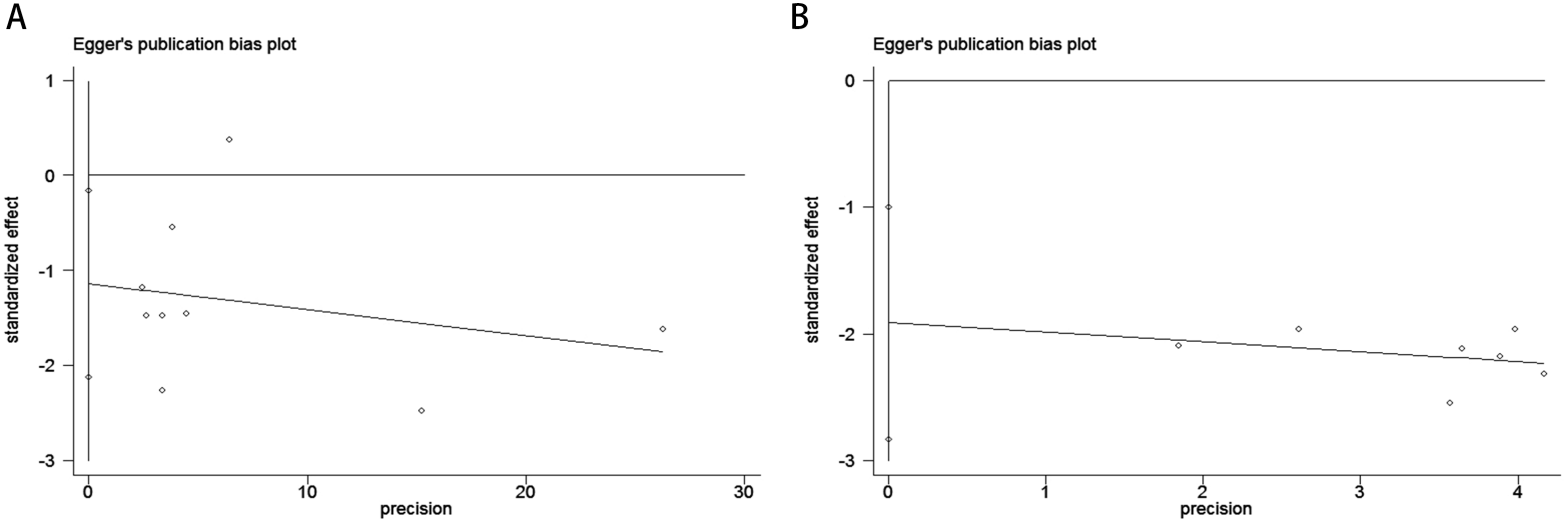
Publication bias assessment. **(A)** Egger’s test for OS (p = 0.504); **(B)** Egger’s test for PFS (p = 0.003).

The Begg’s rank correlation test demonstrated no significant publication bias in the OS analysis of lung cancer patients with comorbid COPD receiving ICIs (p = 0.118 > 0.05; **Figure 4A**). However, the Begg’s test results for PFS revealed significant publication bias in the PFS analysis (p= 0.016 < 0.05; **Figure 4B**). For OS, the results of Egger’s regression test aligned with Begg’s rank correlation test (p=0.504>0.05), further supporting the absence of significant publication bias (**Figure 5A**). In contrast, for PFS, Egger’s test corroborated Begg’s test findings (p=0.003<0.05), confirming the presence of statistically significant publication bias (**Figure 5B**).

### 3.4 Sensitivity analysis

**Figure 6:**
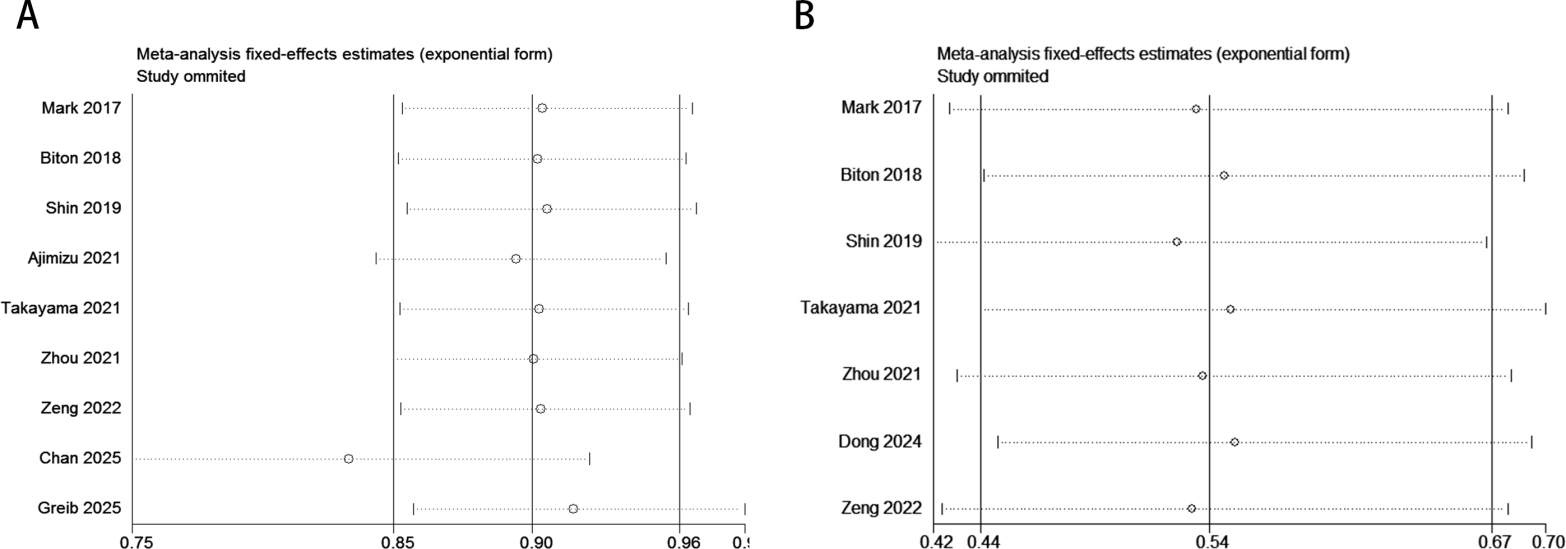
Sensitivity analysis evaluating the robustness of pooled survival outcomes (OS, PFS) in the COPD-lung cancer immunoaxis meta-analysis for patients treated with ICIs. [**(A)**: OS; **(B)**: PFS].

The sensitivity analysis revealed that omitting specific studies (e.g., Chan 2025 or Greib 2025 with HR = 0.98) might significantly reduce the pooled HR for OS, suggesting a dilution effect of these studies on the overall results and potentially weakening the survival benefit conclusion of ICIs in the current meta-analysis (**Figure 6A**). In contrast, for PFS, even after excluding outliers (e.g., Mark 2017 with HR = 0.42), the pooled HR remained stable within the 0.44–0.70 range without crossing the null threshold (HR = 1). All studies consistently demonstrated significant PFS improvement, validating the robust and uniform efficacy of ICIs in delaying disease progression (**Figure 6B**).

## 4 Discussion

Lung cancer and COPD are two of the most prevalent and closely related respiratory diseases worldwide (34, 35). COPD, characterized by persistent airflow limitation and chronic inflammation, is an independent risk factor for lung cancer, with both sharing pathogenic pathways such as smoking exposure and dysregulated immune responses (36). Notably, patients concurrently suffering from COPD and lung cancer exhibit extremely poor prognosis, underscoring the urgency to elucidate their biological interplay. Immune checkpoint inhibitors, which target PD-1/PD-L1 or CTLA-4 pathways to harness the host immune system against malignancies, have revolutionized lung cancer treatment (25, 37). However, significant heterogeneity in clinical responses to ICIs has driven researchers to explore potential biomarkers and comorbidities influencing therapeutic efficacy.

Meta-analysis of OS revealed that lung cancer patients with comorbid COPD had a 10% reduced mortality risk after receiving ICIs (HR = 0.90, 95% CI: 0.85–0.96, p<0.05), with the confidence interval not crossing the null value (HR = 1), indicating statistical significance. PFS analysis showed COPD patients had a remarkable 46% reduction in disease progression risk (HR = 0.54, 95% CI: 0.44–0.67, p<0.001), with the confidence interval far from 1 and minimal heterogeneity (I²=0%, p=0.957), suggesting high consistency across studies. Subgroup analysis further demonstrated that PD-1 monotherapy achieved optimal outcomes for both OS (HR = 0.58) and PFS (HR = 0.52), while PD-1/PD-L1 combination regimens showed weaker benefits, implying monotherapy may be more suitable for COPD populations. Geographical analysis indicated significant PFS benefits in Asia (HR = 0.54), whereas OS did not reach statistical significance (HR = 0.83), potentially due to differences in subsequent therapies or insufficient follow-up. Univariate analysis overestimated COPD’s effect (e.g., OS univariate HR = 0.83 vs. multivariate HR = 0.86), emphasizing the need to control confounders like PD-L1 expression and smoking history. Despite publication bias risks (particularly for PFS), results support that COPD may enhance ICI efficacy through chronic inflammatory microenvironments, though further validation of true effects and optimization of stratification strategies (e.g., COPD subtypes, combined biomarker detection) are needed for personalized treatment. Publication bias analysis revealed critical limitations: OS bias paradox: Begg’s test showed no significant bias (adjusted P = 0.118), but Egger’s test intercept was statistically significant (P = 0.029), suggesting small-sample studies (e.g., Shin 2019 HR = 0.51) might overreport survival benefits, slightly inflating effect estimates. PFS bias significance: Both Begg’s (adjusted P = 0.016) and Egger’s tests (P = 0.003) confirmed substantial publication bias, implying the pronounced PFS benefit (HR = 0.54) may be amplified by selective reporting of positive outcomes. Potential explanations include: (1) Selective publication: PFS, as a secondary endpoint, is more susceptible to “positive result prioritization,” while OS, influenced by subsequent therapies, tolerates negative outcomes better. (2) Endpoint characteristics: OS, confounded by survival status and later-line treatments, may dilute bias effects, whereas PFS, as an early endpoint with subjective measurements (e.g., imaging assessments) and short-term effects, is prone to selective reporting. (3) COPD heterogeneity: Unadjusted confounders (e.g., COPD severity, PD-L1 levels, smoking history) may skew effect estimates. For instance, Ajimizu 2021 (HR = 1.06) suggested certain COPD subgroups (e.g., severe emphysema) might respond poorly to ICIs, though limited sample sizes prevented significant impacts on pooled results. Comprehensive analysis indicates that despite bias risks, both outcomes support COPD’s potential to enhance ICI efficacy via immune modulation: OS: Pooled HR = 0.90 suggests modest survival benefits, though true effects may be slightly weaker, requiring long-term follow-up validation. PFS: HR = 0.54 reflects significant disease control advantages, but publication bias-induced overestimation warrants caution, necessitating expanded datasets to verify robustness. However, this study has limitations: First, COPD diagnosis in included cohorts predominantly relied on pulmonary function rather than imaging parameters, potentially missing emphysema-dominant subgroups. Second, unadjusted confounders like pre-treatment corticosteroid use may overestimate COPD’s independent effects. Finally, correlations between peripheral immune markers and tissue microenvironments require validation via spatial transcriptomics and novel technologies.

In conclusion, the immune interaction between COPD and lung cancer transcends mere pathogenetic linkage and may harbor therapeutic response clues. Redefining COPD as an “immune response modulator” rather than a mere comorbidity could unlock new dimensions in precision immunotherapy. Clinically, COPD may serve as a predictive biomarker for ICI efficacy, informing treatment decisions. Future research should prioritize dose-optimization studies for COPD-lung cancer populations, balancing immune activation and adverse event risks.

## 5 Conclusions

This meta-analysis demonstrates that lung cancer patients with comorbid COPD treated with ICIs exhibit significant clinical advantages in both OS and PFS. Although bias analysis suggests potential publication bias in some results, the overall trends in OS and PFS still support the core conclusion that COPD enhances ICI efficacy through immune-modulatory mechanisms. Future large-scale studies are needed to further validate the robustness of these findings and optimize individualized treatment strategies tailored to COPD subtypes (e.g., emphysema, chronic bronchitis) to balance therapeutic efficacy and safety risks.

## Data Availability

The original contributions presented in the study are included in the article/supplementary material. Further inquiries can be directed to the corresponding author.
